# Carfilzomib-Induced Thrombotic Microangiopathy: A Case Report on a Rare Complication

**DOI:** 10.7759/cureus.85390

**Published:** 2025-06-05

**Authors:** Manogna Pendyala, Ayesha Tahir, Purnoor Kaur, Abhay Shelke, Vinod Khatri

**Affiliations:** 1 Department of Internal Medicine, Mercy Health - St. Vincent Medical Center, Toledo, USA; 2 Department of Hematology Oncology, Bon Secours Mercy Health, Toledo, USA; 3 Department of Pulmonary and Critical Care Medicine, Mercy Health - St. Vincent Medical Center, Toledo, USA

**Keywords:** acute kidney injury, haptoglobin, hemodialysis, hemolytic anemia, lactate dehydrogenase, relapse, schistocytes, thrombocytopenia

## Abstract

Thrombotic microangiopathy (TMA) is characterized by microangiopathic hemolytic anemia, thrombocytopenia, and organ injury due to microvascular thrombosis. Drug-induced TMA is a recognized subtype, but its diagnosis is challenging due to the absence of specific laboratory tests to identify the agent. Many chemotherapeutic agents are associated with TMA. Carfilzomib is a newer chemotherapeutic agent and is an uncommon cause of TMA. We report the case of a 71-year-old female patient undergoing treatment with carfilzomib for refractory multiple myeloma who developed suspected carfilzomib-induced TMA and was managed successfully with plasmapheresis and hemodialysis leading to clinical improvement.

## Introduction

Specific chemotherapeutic agents, such as gemcitabine, oxaliplatin, and vincristine, have been associated with thrombotic microangiopathy (TMA) [1,2]. The latest advances in chemotherapy have significantly improved the prognosis of multiple myeloma. However, most of the patients eventually develop relapsed or refractory disease [3]. Several newer therapeutic agents have been introduced, including carfilzomib, a second-generation proteasome inhibitor approved for use in relapsed and/or refractory multiple myeloma [3]. However, with the increasing use of carfilzomib, carfilzomib-induced TMA has emerged as a potentially serious complication. The pathogenesis is multifactorial, involving the inhibition of the ubiquitin-proteasome pathway and the activation of the alternative complement pathway [4-6]. The onset can vary from weeks to years after initiation of the therapy [7], and management of TMA is challenging as there are no well-established guidelines. Although rare, it is a life-threatening condition that requires prompt recognition and management to prevent significant morbidity and mortality.

## Case presentation

A 71-year-old woman arrived at the emergency department of an outside facility with complaints of diarrhea, difficulty with physical effort, poor appetite, and easy fatigue. Her diarrhea started about four weeks before her visit and was accompanied by a decreased appetite and an increase in fatigue. Her symptoms progressively worsened, affecting her ability to carry out daily activities, which led her to seek further evaluation.

Her medical history was significant for kappa light chain myeloma, diagnosed in 2020 following evaluation of a pathological fracture. A bone marrow biopsy at that time showed 85% plasma cells. She received radiation therapy at multiple sites for bone disease and was initiated on chemotherapy with bortezomib, lenalidomide, and dexamethasone, later transitioning to maintenance therapy with lenalidomide. Initial disease relapse in 2022, as indicated by elevated kappa light chains, led to the initiation of a daratumumab-based regimen. In 2023, due to further relapse, she was started on carfilzomib, cyclophosphamide, and dexamethasone. She completed six cycles of this regimen and was transitioned to maintenance therapy with biweekly carfilzomib and dexamethasone. Her last treatment was administered one week before her current presentation.

On initial evaluation at the outside facility, she was hemodynamically stable, afebrile, lethargic, and not in respiratory distress. Initial laboratory findings revealed acute kidney injury (AKI) and severe thrombocytopenia (Table 1). A respiratory panel tested positive for influenza. She received two units of platelet transfusions and started on Tamiflu and intravenous fluids. Despite supportive care, her renal function deteriorated, prompting nephrology consultation. Given the impending need for renal replacement therapy and concern for TMA, she was transferred to our facility for advanced care.

**Table 1:** Laboratory investigations at the outside hospital BUN: blood urea nitrogen; LDH: lactate dehydrogenase

Lab parameter	At the outside facility	Reference range
Hemoglobin (g/dL)	10.9	12-16
Platelet count (k/uL)	3	130-400
BUN (mg/dL)	38	4-25
Creatinine (mg/dL)	2.1	0.52-1.04
LDH (U/L)	1,087	129-260
Haptoglobin (mg/dL)	81	30-200

At admission to our hospital, she was alert, oriented, and hemodynamically stable, and had no acute distress. On day 2 of admission, she was initiated on hemodialysis due to nonoliguric AKI. Further laboratory findings revealed evidence of hemolysis with elevated lactate dehydrogenase, decreased haptoglobin, and acute anemia. Her complement levels were within the normal range. Additional laboratory tests performed during the hospitalization are listed in Table 2.

**Table 2:** Laboratory investigations at our hospital BUN: blood urea nitrogen; LDH: lactate dehydrogenase

Lab parameter	Day 1	Day 2	Day 3	Day 5	After the first cycle of plasmapheresis	After the last cycle of plasmapheresis	Reference range
Hemoglobin (g/dL)	9	8.3	7.9	7.1	7.5	7.9	12-16
Platelet count (k/uL)	50	26	33	36	69	120	130-400
LDH (U/L)	1,577	1,430	1,478	1,140	808	320	129-260
Haptoglobin (mg/dL)	26	<10	<10	<10	26	<10	30-200
BUN (mg/dL)	56	60	41	27	19	24	4-25
Serum creatinine (mg/dL)	5.3	6.0	4.5	3.8	2.9	2.7	0.52-1.04
Complement C3 (mg/dL)	122	-	-	-	-	-	90-180
C4 (mg/dL)	26	-	-	-	-	-	10-40

**Figure 1:**
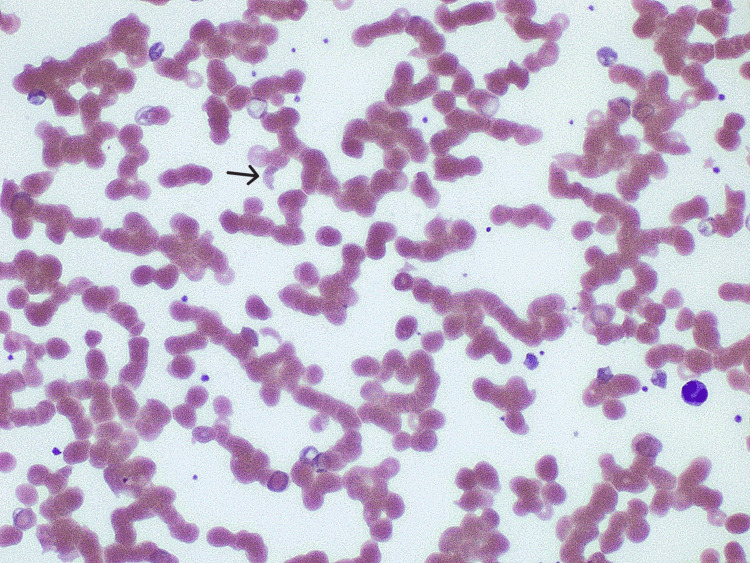
Peripheral blood smear showing schistocytes (black arrow)

Gastrointestinal molecular panels and *Clostridium difficile* toxin tests were negative. A peripheral blood smear showed the presence of schistocytes (Figure 1). Schistocytes are fragmented red blood cells, and their presence indicates hemolysis.

Diagnosis and management

Given the high suspicion for carfilzomib-induced TMA, hematology-oncology was consulted. Other causes of TMA were systematically ruled out. A disintegrin and metalloprotease with thrombospondin type 1 repeats, member 13 (ADAMTS13) activity at 73% excluded thrombotic thrombocytopenic purpura (TTP). Normal complement levels excluded atypical hemolytic uremic syndrome (HUS). Negative Shiga toxin polymerase chain reaction ruled out Shiga toxin-mediated HUS.

The diagnosis of carfilzomib-induced TMA was established. Plasma exchange therapy was initiated on hospital day 6, and she received four sessions. Due to her concurrent influenza infection and clinical improvement with plasmapheresis, anticomplement therapy was deferred. Hemodialysis was discontinued after four sessions; carfilzomib therapy was also discontinued. Her clinical condition improved significantly, and she was discharged with outpatient oncology follow-up.

## Discussion

TMAs are potentially life-threatening conditions characterized by the formation of microvascular thrombi. Various TMA syndromes include TTP, Shiga toxin-mediated HUS, drug-induced TMA, and complement-mediated TMA [1]. Several drugs have been implicated in causing drug-induced TMA, including but not limited to quinine, valproic acid, and chemotherapeutic agents like gemcitabine [2].

Carfilzomib is a second-generation proteasome inhibitor approved by the FDA for relapsed/refractory myeloma [3]. While its increased use has been associated with various adverse effects, carfilzomib-induced TMA is an uncommon complication not described in the original trials [4]. The exact incidence of carfilzomib-induced TMA is unknown. However, the ENDEAVOR trial reported TMA in two out of 463 patients, while the CARDAMON study reported eight cases among 281 patients [5].

The pathogenesis of carfilzomib-induced TMA appears multifactorial. Inhibition of the ubiquitin-proteasome pathway further leads to inhibition of transcription factor nuclear factor kappa B. This leads to the inhibition of the vascular endothelial growth factor pathway and the overproduction of complement, which causes vascular endothelial disruption and the formation of microthrombi [4,5]. Another proposed mechanism is the activation of the alternative complement pathway [6].

The onset of TMA can vary from weeks to years after the initiating carfilzomib therapy [7]. Most cases are reported after the second or third cycle of chemotherapy [6,8], although TMA can develop at any stage. In our patient, TMA occurred after six cycles of carfilzomib, during her maintenance therapy.

Before diagnosing drug-induced TMA, it is essential to rule out other causes like TTP or HUS. ADAMTS13 activity serves as a key differentiator; significantly reduced levels are typical in TTP, while drug-induced TMA often presents with normal or slightly reduced levels. However, treatment decisions are frequently initiated before ADAMTS13 results are available due to the urgency of the clinical situation [8].

Management of carfilzomib-induced TMA primarily involves discontinuation of the offending agent and supportive care [4]. Therapeutic plasma exchange is often initiated early, especially in patients with hemolysis or severe kidney injury. In a comprehensive review involving 114 patients, all six patients who underwent plasmapheresis and received steroids achieved full recovery of renal function and platelet counts [4]. Four plasma exchange sessions resulted in significant improvement in renal function in our case.

Steroids have been suggested in some cases to modulate the immune response. The role of complement inhibition with eculizumab remains unclear, though some case reports have shown successful outcomes with its use, supporting the role of complement in the pathogenesis of carfilzomib-induced TMA [4,7]. A recent case series did not find any effect of eculizumab or steroids in the management of carfilzomib-induced TMA [9]. However, due to limited data, definitive treatment guidelines for carfilzomib-induced TMA are lacking, underscoring the need for further research.

## Conclusions

Carfilzomib-induced TMA is a rare and serious complication associated with the drug and can manifest at any time during the treatment. Early recognition of carfilzomib-induced TMA is of utmost importance to prevent further complications, and this case report spotlights the importance of clinical vigilance and the diagnostic approach to TMA. It should be included in the differential diagnosis in patients with multiple myeloma presenting with worsening anemia and renal failure. Immediate cessation of the offending agent is the mainstay of treatment, along with supportive management. Renal follow-up is recommended in patients with incomplete renal recovery. The role of other management options, including anticomplement therapy and corticosteroids, remains unclear, emphasizing the need for further research.
